# Melt rates in the kilometer-size grounding zone of Petermann Glacier, Greenland, before and during a retreat

**DOI:** 10.1073/pnas.2220924120

**Published:** 2023-05-08

**Authors:** Enrico Ciracì, Eric Rignot, Bernd Scheuchl, Valentyn Tolpekin, Michael Wollersheim, Lu An, Pietro Milillo, Jose-Luis Bueso-Bello, Paola Rizzoli, Luigi Dini

**Affiliations:** ^a^Radar Science and Engineering Section, Jet Propulsion Laboratory, California Institute of Technology, Pasadena, CA 91109; ^b^Department of Earth System Science, University of California, Irvine, CA 92697; ^c^Department of Civil and Environmental Engineering, University of California, Irvine, CA 92697; ^d^ICEYE Ltd., 2150 Espoo, Finland; ^e^College of Surveying and Geo-informatics, Tongji University, 200092 Shanghai, China; ^f^Center for Spatial Information Science and Sustainable Development Applications, Tongji University, 200092 Shanghai, China; ^g^Cullen College of Engineering, University of Houston, Houston, TX 77004; ^h^Microwaves and Radar Institute, German Aerospace Center, 82234 Munich, Germany; ^i^Space Geodesy Centre Giuseppe Colombo, Italian Space Agency, 75100 Matera, Italy

**Keywords:** sea level, Greenland, glaciology, Arctic Ocean, remote sensing

## Abstract

Warming of the ocean waters surrounding Greenland plays a major role in driving glacier retreat and the contribution of glaciers to sea level rise. The melt rate at the junction of the ocean with grounded ice—or grounding line—is, however, not well known. Here, we employ a time series of satellite radar interferometry data from the German TanDEM-X mission, the Italian COSMO-SkyMed constellation, and the Finnish ICEYE constellation to document the grounding line migration and basal melt rates of Petermann Glacier, a major marine-based glacier of Northwest Greenland. We find that the grounding line migrates at tidal frequencies over a kilometer-wide (2 to 6 km) grounding zone, which is one order of magnitude larger than expected for grounding lines on a rigid bed. The highest ice shelf melt rates are recorded within the grounding zone with values from 60 ± 13 to 80 ± 15 m/y along laterally confined channels. As the grounding line retreated by 3.8 km in 2016 to 2022, it carved a cavity about 204 m in height where melt rates increased from 40 ± 11 m/y in 2016 to 2019 to 60 ± 15 m/y in 2020 to 2021. In 2022, the cavity remained open during the entire tidal cycle. Such high melt rates concentrated in kilometer-wide grounding zones contrast with the traditional plume model of grounding line melt which predicts zero melt. High rates of simulated basal melting in grounded glacier ice in numerical models will increase the glacier sensitivity to ocean warming and potentially double projections of sea level rise.

## Significance

We present a record of glacier ice dynamics and ice melt rate at the boundary between grounded ice and ocean—or grounding line—of Petermann Glacier, a major outlet glacier in Northwest Greenland. The traditional view of grounding lines implemented in ice sheet models in charge of projecting sea level rise is that they not migrate during the tidal cycle and experiences no ice melt. Instead, the satellite record reveals kilometer-size grounding line migrations—or grounding zones—with preferential intrusions along preexisting subglacial channels. The highest melt rates of ice are recorded within the grounding zone. Vigorous ice-ocean interaction in kilometer-wide grounding zone will make projections of sea level rise from glaciers potentially double.

The Greenland Ice Sheet has lost billions of tons of ice to the oceans in the last few decades, increasing global sea level by 14 mm since 1972 (1). The mass loss is a combination of enhanced surface melt from warmer air temperatures and enhanced ice discharge at the ice sheet periphery from warmer ocean temperatures, with little contribution from changes in snowfall accumulation. Most ice loss has been caused by the warming of the subsurface ocean waters around Greenland, which melted the glacier interface with the ocean and forced the retreat (2–4), with additional feedback from a weakening ice melange at glacier fronts (5). In the case of glaciers extending into floating ice shelves that do not calve immediately in the ocean as icebergs, the glacier evolution has been more challenging to observe and analyze, but the physical processes driving the mass loss are likely to be similar (6). Warmer ocean waters that erode grounded ice reduce basal resistance to flow, the glaciers accelerate, and sea level rises. Most ice sheet models employ parameterization of ice melt that assumes zero melt at a grounding line that does not migrate with tides (7, 8). It has been especially recommended not to apply melt in model mesh elements that cross the grounding line (9).

Prior modeling studies have questioned the assumption of a grounding line with zero melt. Parizek et al. (10) used a coupled glacier–ice shelf-ocean plume model that included oceanic influence over multiple kilometers upstream of the grounding line instead of hundreds of meters. Indirect evidence available at the time included bright reflections from radar sounding echoes upstream of the grounding line that were typical of ice over seawater extending inland over several kilometers and gradually fading to weaker reflections typical of ice over a thin layer of freshwater (11). Model simulations indicated that ice melt applied to kilometer-size grounding zones would reduce glacier stability and double the mass loss compared to that from standard simulations. Walker et al. (12) developed a flexural model of elastic ice on an elastic bed that suggested the presence of kilometer-scale intrusion of seawater at tidal frequencies along subglacial water channels upstream of the grounding line caused by variations in ice flexure. More recently, Robel et al. (13) simulated layered seawater intrusion under grounded ice and estimated that such a configuration would increase the mass loss by a factor of up to two. Despite these modeling studies, the physical processes driving melt under the grounded portion of an ice sheet are still represented in their simplest forms in many ice sheet models, i.e., a grounding line with no melt (7).

A number of recent studies using radar interferometry data have recently pointed out the existence of broad, kilometer-size “grounding zones” in Antarctica, e.g., on Pine Island Glacier (14), Thwaites Glacier (15), Getz Ice Shelf (16), and other parts of Antarctica. Note here that grounding zone refers to the zone of migration of the grounding line during the tidal cycle. It should not be confused with the zone of tidal flexing of an ice shelf, previously called a grounding zone (17, 18), but more appropriately named the tidal flexure zone. In the case of Thwaites Glacier, thinning rates observed from a time series of TanDEM-X DEMs suggested high rates of basal melt in areas of recent ungrounding of glacier ice (15), but the study did not address basal melt rates within the grounding zone itself in much detail and at different epochs.

**Fig. 1.**
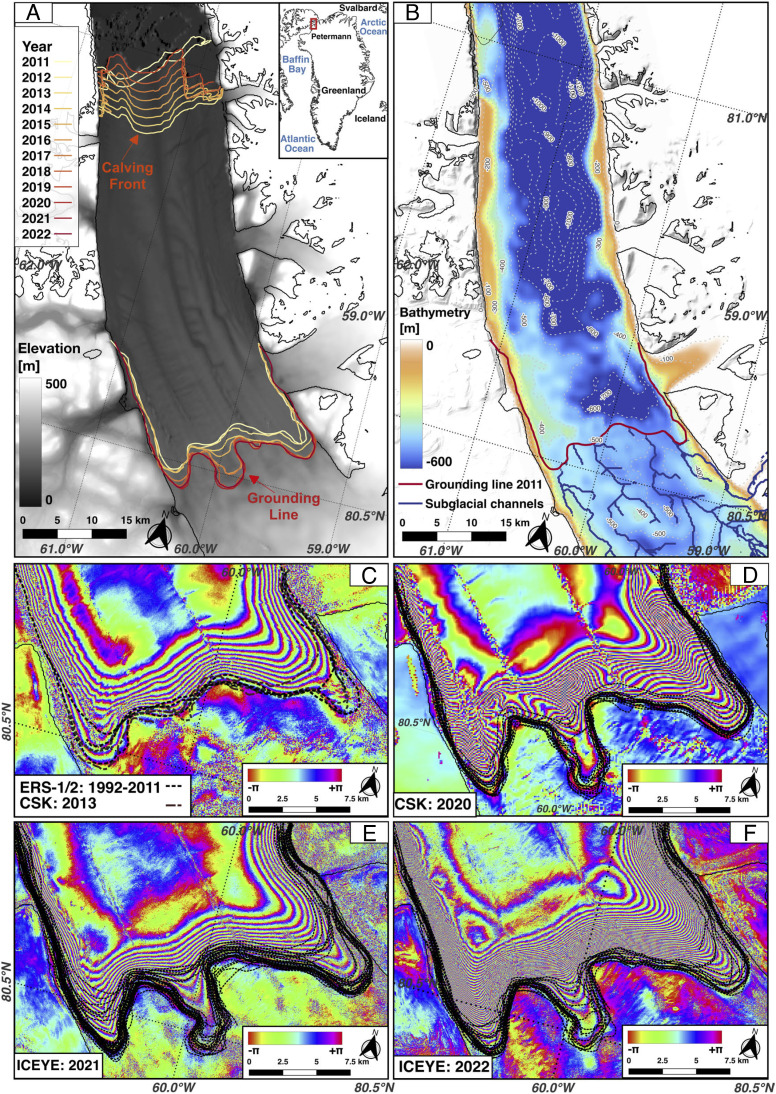
Petermann Glacier, North Greenland (80° 45′ N, 60° 45′ W); (*A*) grounding line and ice front positions color-coded from 2011 to 2022 overlaid on surface topography from TanDEM-X. Ice motion is from south to north (*Bottom* to *Top*) with floating ice in the north. The *Top*-*RightInset* shows the glacier’s location. (*B*) Bathymetry from BedMachine Greenland Version 5 on grounded ice and from a 3D inversion of Ocean Melting Greenland gravity data on floating ice color-coded from 0- (brown) to 800-m depth (dark blue), with 100-m depth contours, grounding line position in 2011 (red), and subglacial channels calculated using a water flow model (dark blue). Differential radar interferograms and corresponding grounding line positions from (*C*) 1992, 1996, and 2011 from ERS-2 and 2013 from CSK; (*D*) 2020 from CSK; (*E*) 2021 and (*F*) 2022 from ICEYE. Each full cycle of the interferometric phase represents a 3.1-cm vertical motion of the ice surface with ERS-2 and 1.8 cm with CSK and ICEYE.

In this work, we employ a time series of dense, high-resolution observations of ice motion and ice surface elevation to document the grounding line variability of Petermann Glacier, a major marine-terminating glacier in Northwest Greenland. Petermann drains an area of 74,348 square km with an ice volume above sea level equivalent to a 38-cm global sea level rise equivalent (SLE) (Fig. 1). With its neighbor Humboldt Glacier (19 cm SLE) (19), two ice streams in Northeast Greenland (6) (116 cm SLE), and Jakobshavn Isbrae (48 cm SLE) (20), they form the largest threat for rapid sea level rise from Greenland in the coming decades because their drainage basins are grounded below sea level (21). Until recently, Petermann has been close to a state of mass balance, with a cumulative loss of 56 Gt (1 Gt = 10^12^ kg) for the period 1972 to 2017 (1). Its floating ice shelf, one of the longest in the northern hemisphere, was reduced by one-third during a series of large calving events in 2010 and 2012. Around 2016, the glacier grounding line started to retreat at about 1 km/y at the center, which was unprecedented this century (22). The retreat has been attributed to warmer ocean and air temperatures starting in 2016 (23).

In prior studies, we used less differential interferometric synthetic aperture radar (DInSAR) data to map the grounding line with high precision (24): two DInSAR from the European Remote-Sensing Satellite (ERS) 1-2 data acquired at C-band frequency (5.6 cm wavelength) in 1992 on a 3-d cycle, two in 1996, one in 2000, and one in 2011. We added two more from the Italian Cosmo-SkyMed (CSK) constellation acquired at X-band (3.1 cm wavelength) at a 1-d time separation 16 d apart in 2013. In 2014, the European Union launched the C-band Sentinel-1a (S1) on a 12-d repeat cycle, followed by S1b in 2015 placed on a 6-d repeat cycle with S1a. S1 provides the first continuous stream of DInSAR data on a 6-d repeat cycle. In 2020, we started systematic acquisitions of CSK data with 1-d separation pairs at a 16-d repeat cycle. In 2021, the Finnish ICEYE X-band constellation (25) acquired data continuously on a 1-d repeat cycle.

**Fig. 2.**
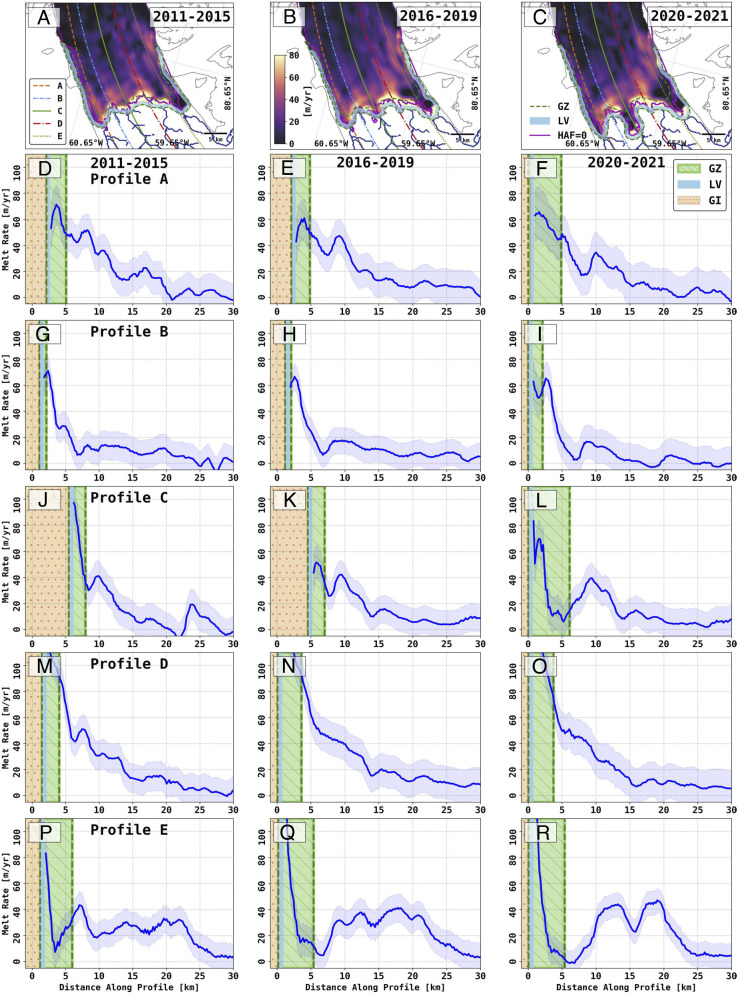
Ice melt rates in meters per year on the floating ice of Petermann Glacier, Greenland for (*A*) 2011 to 2015; (*B*) 2016 to 2019; and (*C*) 2020 to 2021 with grounding zone (GZ, dotted black), limit of viability (LV, blue band), line of flotation (height above floatation, HAF, equal to zero, purple), and subglacial channels (black on grounded ice). (*D*–*R*) are melt rates calculated along profile (*D*–*F*) A, (*G*–*I*) B, (*J*–*L*) C, (*M*–*O*) D, and (*P*–*R*) E. Profile locations are color-coded in (*A*). In (*D*–*R*), the green forward diagonal-hatched column is the grounding zone (GZ), the vertical light blue band is LV, and the orange dotted-hatched column is grounded ice (GI).

To map the ice sheet surface elevation, we use a monthly time series of digital elevation models (DEM) from the German Space Agency (DLR) TanDEM-X SAR mission for the period 2011 to 2021 (26) (Fig. 1). In combination with ice velocity and surface mass balance, these data are employed in a Lagrangian mass conservation framework to calculate ice shelf melt rates (27); Fig. 2; *Materials and Methods*. Previous attempts at mapping ice shelf melt rates using optical-derived DEMs conservatively stopped several kilometers seaward from the grounding line to be sure to remain in a region of hydrostatic equilibrium (28). Here, we delineate the grounding zone, i.e., a region of tidal migration of the grounding line caused by seawater intrusion, and we calculate basal melt rates in the grounding zone (*Materials and Methods*) after verifying that hydrostatic equilibrium still applies in that zone. We document how the grounding zone evolves from the 1990s to the present and observe how the melt rates change during the retreat. We conclude on melt rates in the grounding zone before and during the retreat and discuss the impact of the results on projecting glacier evolution in a warming climate.

## 1. Results

The 1992 to 2011 grounding zone (GZ) of Petermann is broadly rectilinear with two extension lobes on each side (Fig. 1). The GZ width is 1.5 km at the center in 1992, 1996, 2011, and 2013 and 3 to 4 km on the sides. In 2016 to 2018, the GZ started to retreat and widened to 6 km at the center, 6 km on the sides, and 1.7 km elsewhere. In 2019 to 2021, the GZ width increased to 6.5 km at the center and on the sides and 2 km elsewhere (Figs. 1 and 2). For comparison, if we calculate the migration of the grounding line based on hydrostatic equilibrium, bed slope, surface slope, and tidal height as in ref. 29, we find a GZ width of 200 m, i.e., more than one order of magnitude smaller. A multikilometer-wide GZ is therefore not expected from hydrostatic considerations alone.

After calibration of the DEMs, we find that the height above flotation (HAF) of the ice surface is zero at the center of the inferred GZ and less than 2 to 3 m within the GZ, which is consistent with the hypothesis that ice is floating in the GZ. From 1992 to 2013, 2011 to 2015, 2016 to 2017, 2020 to 2021, the GZ area grew in size from 74.5, 86.7, 81.9, and 125 km^2^, respectively; that is, it nearly doubled. Most of the change took place at the glacier center and along the western and eastern extension lobes.

The GZ retreated along an area of retrograde slope, i.e., where bed elevation drops in the inland direction (22). The retreat is less apparent along the extension lobes, where the bed slope is prograde. From 2017 to 2022, the GZ retreated 1.6 km on the western lobe, 3.7 km at the glacier center, and near zero elsewhere. From 2019 to 2022, the seaward end of the GZ remained at the same location as in the 1990s, but the upstream limit migrated upstream. In 2022, the GZ migrated less frequently to its most seaward position at the glacier center, which indicates that the new cavity was almost always open.

**Fig. 3.**
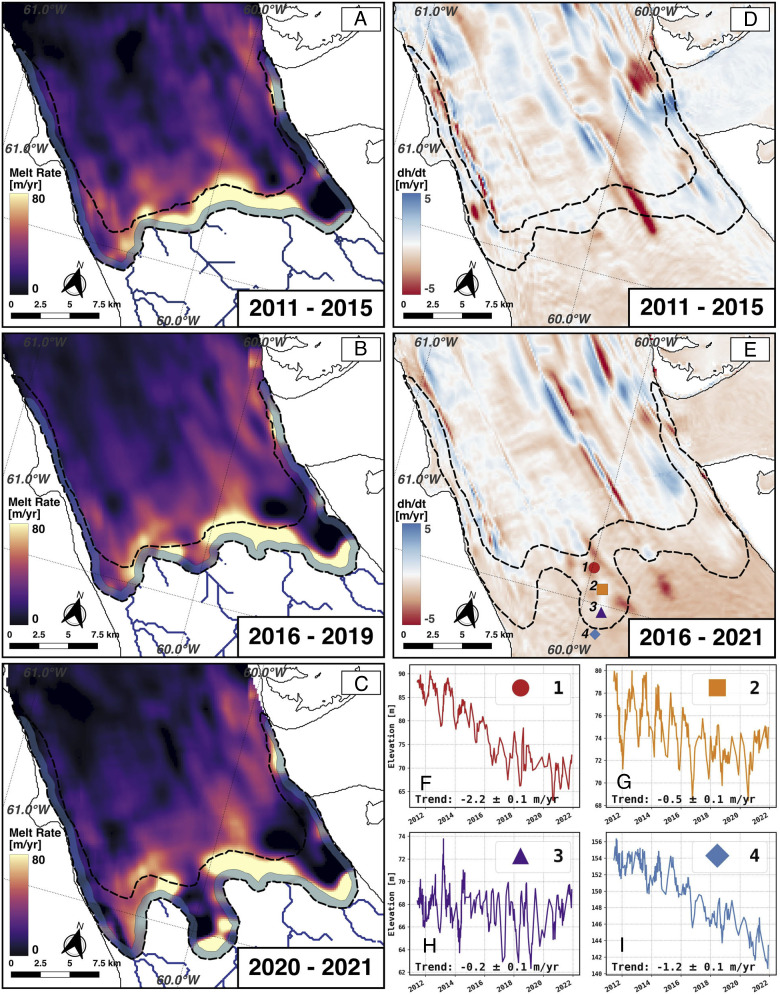
Ice melt rates near the grounding zone of Petermann Glacier in (*A*) 2011 to 2015; (*B*) 2016 to 2019; and (*C*) 2020 to 2021 with the position of the grounding zone (dotted line) and limit of viability (blue) for the calculation of melt rates where ice is floating. (*D*) Cumulative change in surface elevation from 2011 to 2015 color-coded from −5 m/y to +5m/y, and (*E*) similar results for 2016 to 2021. (*F*–*I*) Time series of surface elevation at points # 1 to 4 to document the ice surface evolution during the retreat on floating ice (points # 1 to 3) vs. grounded ice (point # 4).

Using a subglacial water flow model, we predict four principal channels of subglacial water discharge for the glacier (Fig. 2) (*Materials and Methods*). The main channel (Profile *C* in Fig. 3) drains 66% (or 11 m^3^/s on average) of the total subglacial water discharge in winter from geothermal and frictional heat versus 27% for the secondary channel (Profile *D*) and 3% for each of the other two channels (Profiles *A* and *E*). In summer (June to August), the main channel drains 43% of the total water discharge (or 86 m^3^/s on average for the three summer months) versus 45% for the secondary channel, and 6% each for the other two channels. The region of the most pronounced retreat is therefore aligned with the main channel, but we do not observe a similarly large retreat along the secondary channel, which discharges almost as much subglacial water. Over the time period 2001 to 2022, subglacial water discharge increased from 149 to 301 m^3^/s in summer. This doubling in subglacial water discharge must have enhanced the ice melt rates but cannot be the only factor contributing to the retreat (Figs. 1 and 2).

The melt rates deduced from the time series of TDX DEMs using a Lagrangian approach are consistent with those retrieved with an Eulerian approach (*SI Appendix*, Fig. S1) but with improved precision. The highest melt rates are recorded in the GZ, not farther out on the ice shelf. As predicted by the plume model, we observe a secondary peak about 10 to 15 km downstream. Melt rates, however, average 60 ± 15 m/y at the center of the GZ and exceed 80 to 100 ± 15 m/y along the east and west lobes (Fig. 2). The results for 2011 to 2021 do not change if we use different degrees of spatial smoothing (500, 750, and 1,250 m) and longer time separation (2 to 3 y) between DEMs instead of 1 y.

We find a marked evolution of the melt rates in the newly formed cavity at the center. Initially, the melt rates averaged 60 ± 15 m/y (Fig. 2*A*). As the cavity formed and the grounding line retreated, the average melt rate dropped to 40 ± 11 m/y (Fig. 2*B*) until 2020, at which point the upper part of the cavity experienced average melt rates again in the range of 60 ± 15 m/y (Fig. 2*C*).

When we integrate the melt rate calculated over the entire ice shelf, we find a mass flux of 12.0 ± 2.8 Gt/y in 2011 to 2015, 15.6 ± 2.7 Gt/y in 2016 to 2019, and 14.6 ± 2.9 Gt/y in 2020 to 2021. These values indicate a 20 to 30% increase in ice melt since 2011 to 2015. The steady-state grounding line flux of the glacier is 10.5 ± 0.7 Gt/y (1); hence, ice melt by the ocean exceeds the glacier balance flux by 14% in 2011 to 2015 and 39% in 2020 to 2021; that is, the ice shelf was losing mass during those times, and the mass loss accelerated after 2015. If we convert the mass flux in Gt/y to a freshwater flux in Sverdrup (1 Sverdrup = 1,000,000 m^3^/s and 5 Gt/y is 0.16 mSv), the total freshwater flux increased from 0.38 mSv in 2011 to 2015 to 0.50 mSv and 0.46 mSv in 2016 to 2017 and 2020 to 2021. For comparison, the freshwater flux for the entire ice shelf has been previously estimated at 0.33 mSv in year 2011 (30), which is consistent with our remote sensing data.

Within the GZ, we calculate a total melt flux of 3.7 ± 0.7, 4.9 ± 0.7, and 6.1 ± 0.8 Gt/y from 2011 to 2015 to 2016 to 2019 and 2020 to 2021, respectively, or 30% to 40% of the total ice shelf meltwater. The GZ therefore plays a major role in total ice shelf melt despite its relative small area.

From the differential displacement of the ice surface during the tidal cycle, which is mostly a vertical motion, we indirectly document the height of the water column that infiltrates beneath the glacier ice at high tide. In the early part of the record (2016 to 2019), the water height varies almost linearly from the full positive tide (2 m at the entrance for a ± 1-m tide) at the seaward edge of the GZ to zero at the upstream edge. This height is much thinner than further downstream, outside of the GZ and outside of the flexure zone, where it rapidly increases to several hundred meters (Fig. 1). The water layer that infiltrates beneath grounded ice during the tidal cycle is only of the order of a meter.

For a tidal amplitude of ±1 m, the cavity will fluctuate from 0 to 2 m at the entrance and taper linearly down to zero at the end, which is equivalent to an average water thickness of 1 m. The water volume in the cavity will therefore change by 0.075 km^3^ in 2011 to 2015 and 0.125 km^3^ in 2020 to 2021 within 6 h. If we assume a water temperature *T* = 0.3 °C at 500-m depth in the cavity (23), a freezing point of seawater *T*_*f*_ = −2.3 °C at that depth, and a salinity of *S* = 35 psu, we have a thermal forcing, (*T* − *T*_*f*_), of 2.6 °C. Using a heat capacity of cold seawater, *C*_*p*_ = 3,974 J kg^−1^ °C^−1^, and converting the water volume to a mass transfer, *m*, we obtain an ocean heat transfer, *Q* = *mC*_*p*_(*T* − *T*_*f*_) of 0.36 10^11^ W to 6.0 10^11^ W to the ice. If all the ocean heat is available to melt ice with a latent heat of fusion, *L*_*f*_ = 334,000 J/kg, this yields a freshwater flux increasing from 0.11 mSv in 2011 to 2015 to 0.18 mSv in 2020 to 2021 or a mass flux of 3.5 to 5.7 Gt/y. These estimates, which assume that the warmest ocean waters can reach the GZ, are consistent with melt rates deduced from remote sensing data, hence providing additional confidence in the results.

We analyze the cumulative thinning of points within the GZ (Fig.3*D*–*I*). Point #1, at the seaward edge of the GZ, thinned at 2.2 ± 0.1 m/y vs. 1.2 ± 0.1 m/y on grounding ice at point #4. Since point #1 is fully afloat, the surface thinning of 22 m in 10 y translates into a total thickness reduction of 204 m. In contrast, point #4 remains grounded during the entire period, hence lost 12 m of thickness. If we attribute this ice lowering to dynamic thinning, i.e., a longitudinal stretching of grounded ice caused by ice flow acceleration, the same rate will apply to the proximate floating ice. With that correction, we deduce that ice shelf melt alone must have removed (204 − 12) m = 192 m of ice in 10 y at point #1 for a total cavity height of 204 m, which is a rapid rate of ice removal by Greenland and Antarctic standards. At points #2 and #3, in the middle of the cavity, melting is accompanied by thinning until 2019, followed by a small amount of thickening in 2019 to 2021. We suspect that the bending stresses, which push ice below hydrostatic equilibrium immediately seaward of the grounding line, relaxed as the grounding line retreated, which resulted in a gain in surface elevation instead of an expected drop in elevation caused by ice melt (12). A similar evolution of the ice surface was noted on Thwaites Glacier, Antarctica, during its retreat: The ice surface elevation increased during the retreat downstream of the GZ, while radar sounders indicated a reduction in total ice thickness (15). As bending stresses are not included in our calculation of ice melt, our remote sensing results may underestimate the true magnitude of ice melt during the retreat. With bending deflections in the range of 1 to 2 m, the uncertainty in melt is in the range of 9 to 18 m/y.

## 2. Discussion

Our results have vast implications for the modeling of glaciers terminating in ocean waters. Prior attempts at estimating melt rates using DEMs from optical data excluded the area within 5-km downstream of the grounding line and reported lower melt rates with peak values of 50 m/y at Petermann Glacier (28). Here, we are able to extend these calculations to the grounding zone because we document the full extent of the grounding line migration and verify with precision DEMs that the ice surface remains close to flotation in the GZ. We exclude from our calculations a narrow band where the ice transits from grounded to floating within the time separation of the DEMs, which is one year (limit of viability about 700 m in width or 1/2 y of ice motion in Figs. 2 and 3). The results reveal that the melt rates continue to increase within the GZ and are, in fact, the highest anywhere on the ice shelf. We are confident that the vertical motion of the ice in the GZ is caused by seawater intrusion and not, for instance, by flexural bending upstream of a fulcrum located at a fixed grounding line because such ice motion above the fulcrum would be of the opposite sign compared to the tide (12).

The traditional plume model for ice shelf melt (31, 32) predicts peak melt about 10 to 15 km from a fixed grounding line, which is not observed here. The grounding line migrates over considerable distances in response to tidal forcing, which brings a large amount of ocean heat, at high speed, beneath grounded ice. The speed of transfer may be approximated by the width of the GZ divided by the tidal cycle, or 6 h. Thermodynamics dictates that the ice melt rate is the product of the heat capacity of water, *C*_*p*_, times the ocean thermal forcing, *T* − *T*_*f*_, and the entrainment speed, *e*, of the water along the ice boundary. The entrainment speed of pressurized water in the GZ is the rate of opening of the cavity, i.e., 6 km in 6 h, or 28 cm/s. Such a water speed matches peak values observed in meltwater plumes of Greenland tidewater glaciers (33) and ice shelf channels beneath Petermann Ice Shelf (34). The high flow speed will be conducive to vigorous melt and justify a posteriori high melt rates in the GZ. This situation contrasts with zero melt at fixed grounding lines used in some ice sheet models. Other ice sheet models use depth-dependent melt parameterizations (7) which predict the largest melt at the grounding line. These models however differ in how they implement melt on elements that cross the grounding line (9). Models that have zero melt on model elements crossed by the grounding line, called No-Melt Parameterization (NMP), have been recommended by ref. 9, but these models are not consistent with our observations. As suggested by refs. 8–10 and 13, the implications of wet, broad grounding zones for sea level rise projections are significant, especially as more groups adopt full-melt parameterization (FMP) or subelement melt (SEM) for representing basal melt grounding lines as in ref. 35.

Tidal flushing in kilometer-size GZ will progressively reduce basal resistance to flow if the ocean gets warmer. A prior study of ephemeral grounding of ice shelves revealed that the effect is most efficiently felt once the ungrounding of grounded ice is permanent during the tidal cycle (36). The cavity that developed at the center of Petermann’s GZ over the time period 2016 to 2022 is about 20 square km in size. Assuming a spatially averaged basal drag of 1 bar or 100 kPa (Pascal), the ungrounding removed a force of 2TN (Tera Newton). For comparison, assuming a uniform lateral drag of 1 bar along the ice shelf sides (two sides) and an ice shelf thickness of 500 m, the removal of a 20-km long ice shelf would reduce the buttressing force by the same 2 TN. Hence, the grounding line retreat at the glacier center is equivalent to the hypothetical removal of the entire ice shelf in terms of changes in buttressing force. The loss of basal resistance yielded a readjustment in stress balance at the grounding line region and beyond, which was accompanied by a glacier speed up of 15% between 2015 and 2021 (22).

Several modeling studies have indicated that a wide GZ with high melt rates could double the projections of glacier loss (10, 12, 13, 37). This increase in ice sheet sensitivity may help explain the inability of previous models to reproduce rapid rates of sea level rise during past warm periods (38) and the generally too-low ice loss simulated during the recent historical period by ISMIP models (39).

Our results demonstrate that high melt rates should be applied in kilometer-size GZ instead of zero melt on Petermann Glacier. Several explanations are possible for a wide GZ. One is the presence of bending stresses (12), which relax as the grounding line retreats and enable greater penetration of seawater at the GZ wedge. Second, seawater is applied under pressure (40), which violates the assumption that water is at the hydrostatic pressure. Third, the bed may be deformable (41, 42), which facilitates seawater intrusion over greater distances. Fourth, seawater may propagate in the till or through preexisting subglacial channels (11). Fifth, simulations of grounding line migration as the propagation of an elastic crack suggest the possibility of kilometer-size GZ (43). Finally, bed topography is not uniform beneath the glacier and may include roughness elements that will facilitate the infiltration of seawater at high tide or conversely will trap seawater intrusion at low tide (42).

Prior simulations of ice shelf melt beneath Petermann did not produce high melt rates (44), but the model resolution was 20 m in the vertical dimension and did not include a GZ. It would be of interest to extend the modeling of ice shelf melt with vigorous tidal flushing over a thin (about 1 m) GZ multiple kilometers in width. It will also be of interest to confirm these high ice melt rates in situ, e.g., using portable, coherent, radar sounding devices (45).

The glacier configuration of Petermann is not unique to Greenland. It may be representative of many other glaciers terminating into an ice shelf in other parts of Greenland and to glaciers and ice shelves in Antarctica. Wide, heterogeneous GZs have already been revealed in Antarctica (14–16). Wide GZ may be exposed to high melt rates from vigorous tidal flushing. Conversely, narrow GZ may experience limited tidal flushing and ice melt, as in ref. 46, or seawater may be trapped at low tide (47), which will limit heat exchange and ice melt. We need to investigate these physical processes in more detail. To model the access of warm waters to the cavity, we also need to resolve the detailed shape of the ice shelf cavities, which is poorly known. While progress has been made with airborne gravity data, data are needed to constrain the gravity inversion and refine the details. At present, the inferred cavity is asymmetric east–west and includes a ridge about 10 km from the grounding line. The depth of the ridge is affected by uncertainties of ±60 m, which need to be lowered to improve our understanding and modeling of the ocean circulation beneath the ice shelf.

## 3. Conclusions

Using a dense time series of satellite observations of ice surface elevation and millimeter-scale vertical motion of ice as it reaches flotation at tidal frequencies, we find that the grounding line of Petermann Glacier migrates over a zone considerably wider than anticipated, i.e., 2 to 6 km instead of a few 100 m, while deviating little (2 to 3 m) from hydrostatic equilibrium. Using a Lagrangian approach with precision DEM data, we find that the GZ experiences the highest melt rates anywhere on the ice shelf, with values averaging from 60 ± 15 m/y at the glacier center to 80 to 100 ± 15 m/y at the glacier sides. During the retreat and formation of a new cavity, the average melt rates dropped to 40 ± 11 m/y before increasing again to 60 ± 15 m/y as the cavity became fully formed. In light of these results, we recommend revisions in the representation of ice melt rates at grounding lines in numerical ice sheet models. We suggest that ice sheet models adopt subshelf melt schemes that include melting on elements at and upstream of the grounding line (13). Separately, this modification will cause adjustments of the basal friction at and upstream of the grounding line, which has implications for glacier sensitivity to forcing (43). The revisions will likely produce higher projections of mass loss from glaciers and higher rates of sea level rise from ice sheets, possibly by up to a factor of two. We recommend further studies to examine the physical processes taking place in the grounding zone due to its critical role in glacier evolution.

## Materials and Methods

### A. Digital Elevation Models.

We use 302 digital elevation models (DEM) from the TanDEM-X (TDX) mission generated by the German DLR for the period 2011 to 2021 on a monthly basis. The data are automatically calibrated at high elevation using ICESat-1 data (26) and assuming no change in elevation at those locations. We reference the TanDEM-X DEM data (1.3-m error) to mean sea level using the EIGEN-6C4 geoid model (0.1- to 0.4-m error) (48). We correct the data for Mean Dynamic Ocean topography from the MDT-CNES-CLS18 dataset (0.2-m error) (49), changes in ocean tides from the Arctic Ocean Tidal Inverse Model-5 km (AOTIM5) (50, 51) using PyTDM (52), and changes in atmospheric pressure calculated from hourly mean sea level pressure from the fifth-generation global reanalysis from the European Centre for Medium-Range Weather Forecasts ERA5 (0.1-m error for tide and pressure) (53). A small bias in absolute elevation is estimated and removed at the ice–ocean boundary to ensure that mean sea level averaged zero m. Ice shelf surface elevation is translated into ice thickness using a density of 0.917 g/cm^3^ for ice and 1.028 g/cm^3^ for seawater. A height above flotation is calculated assuming that thickness minus free-board height times water density equals thickness times the density of ice. Thickness of grounded ice is surface elevation from TDX minus bed elevation from BedMachine.

### B. Ice Velocity.

We use measurements of annual ice velocity from European Space Agency (ESA)’s Earth Remote Sensing (ERS) 1 and 2, Envisat ASAR, Sentinel-1 and 2, NASA’s Landsat 7 and 8, Canadian Space Agency (CSA)’s RADARSAT-1, Japanese Space Agency (JAXA)’s ALOS PALSAR, and the United States Geological Survey (USGS)’s Landsat-4/5 (1, 22). Velocities are used to track ice blocks with time in the TDX DEMs.

### C. Grounding Zones.

We use prior mappings of the grounding line from synthetic aperture radar interferometric data (24) acquired in 1992, 1996, 2000, and 2011 at a 3-d cycle by ERS-1/2 (1-d in 1996 and 2000), and 2013 with a 1-d time separation on a 16-d repeat cycle by the Italian Space Agency (ASI)’s Cosmo SkyMed (CSK). We lump these 8 differential interferograms together to define a GZ prior to 2014. From 2014 to present, and repeating the exercise on an annual basis, we use ESA’s Sentinel-1 on a 6-d repeat cycle, CSK data at a 1-d repeat cycle acquired on a 16-d repeat cycle in year 2020, and Finish ICEYE data at a 1-d repeat cycle in years 2021 to 2022 (25).

### D. Bathymetry.

The surface and bed topography of Petermann is from IceBridge BedMachine Greenland, Version 5 (BMv5) (54). The surface elevation is the Greenland Ice Mapping Project (GIMP) DEM from years 2007 to 2008 (55); bed elevation is deduced from GIMP using ice thickness measurements conducted by the University of Kansas since the early 1990s. Fjord depth includes multibeam echo sounding at the ice shelf front (56) part of the International Bathymetry Chart of the Arctic Ocean 408 V3 (IBCAOv3) (57), BMv5 on grounded ice, and a novel bathymetry derived from a three-dimensional inversion of airborne gravity data in between. The airborne gravity data were collected on September 7 to 13, 2020, by NASA’s Earth Venture Mission-2 Ocean Melting Greenland (OMG) using the Sanders Geophysics Ltd. AIRGrav instrument deployed on a Cessna Grand Caravans 208B. The survey was conducted at 110 knots, with a ground clearance of 150 m. The data were filtered with a 1-km half-wavelength filter with a RMS error of 1.5 mGal. We employ the Geosoft GM-SYS 3-D with Parker’s method to minimize the misfit between calculated and observed gravity. The model domain has three horizontal layers at a grid spacing of 500 m: 1) a solid ice layer with a density of 0.917 g/cm^3^, 2) an ocean water layer with a density of 1.028 g/cm^3^, and 3) a rock layer with a density of 2.67 g/cm^3^. A forward model of the gravity is calculated using the bed elevation from BMv5 and IBCAOv3. We calculate the direct current (DC) shift between modeled and observed gravity in areas where bed elevation is known from conductivity, temperature, and depth (CTD), BMv5, or multibeam echo sounding data. We interpolate the DC shift onto a regular grid using a minimum curvature algorithm, correct the observed gravity with the interpolated DC shift, fill the data gaps with the model results, and invert the resulting gravity field where bed elevation is not known. The interpolation of the DC shift accounts for natural variations in underlying geology across the model domain caused by variations in crustal thickness, sedimentary basins, or intrusions. The inversion stops when the misfit between observed and modeled gravity is less than 0.1 mGal, which corresponds to a nominal error in sea floor depth of ±60 m. The inversion reveals the presence of a ridge 10 km from the 1996 grounding line, which was previously reported using a two-dimensional inversion of coarser resolution, sparser sampling, NASA’s Operation IceBridge gravity data (58).

### E. Subglacial Channels.

We use a D-infinite channel model (59) to model the distribution of subglacial channels beneath the grounded ice of Petermann Glacier at 150-m spacing, which is the spacing of BedMachine. Subglacial water discharge includes the contribution of ice melt from a geothermal flux of 51 mW/m^2^ (milli-Watt per square meter), basal friction assuming full sliding of the glacier at the bed, using the ice surface velocity, and assuming that 50% of the available heat is used to melt ice. The calculation is repeated with the addition of summer runoff from the Regional Atmospheric Climate Model Version p2.3 (RACMOp2.3) (60) for years 1958 to 2021 assuming that runoff produced at the surface is immediately transmitted to the bed directly below via natural conduits. Subglacial meltwater discharge is calculated in cubic meters per second on a monthly basis for the time period 1958 to 2022.

### F. Ice Melt Rates.

Two approaches are possible to express mass conservation on floating ice shelves and deduce the rates of bottom melt (27): 1) an Eulerian approach calculates melt rates at point locations from mass conservation (27). This approach requires spatial smoothing to mitigate the impact of heterogeneity in ice thickness moving with the glacier. We use a spatial filter of 750 m; 2) a Lagrangian approach calculates the melt of ice blocks as they travel down the glacier from mass conservation (27), which removes the impact of heterogeneity in ice thickness. A time separation of one year is desirable to estimate melt rates with precision. Both approaches assume that ice is in hydrostatic equilibrium. Ice is in hydrostatic equilibrium within the GZ because the ice surface is lifted up at high tide from seawater intrusion, and the ice surface is within 2 to 3 m of flotation (Fig. 2). For one-year time separation between DEMs, we eliminate a “limit of viability” zone in our estimates to exclude ice elements that were fully grounded during the integration period, i.e., a zone equal to half the annual displacement in width, or 700 m (Fig. 2). We combine the elevation changes from the DEMs with surface mass balance from RACMOv2.3p (0.1 m/y error) (60). Ice tracking and flow divergence are calculated using annual ice velocity (1 m/y error). Particle trajectories are updated on a monthly basis from the annual velocity mosaics. Each basal melt rate estimate is attributed at the particle path midpoint. We compute annual elevation changes from DEMs acquired in consecutive years during the same month to obtain a uniformly spaced sampling. For yearly melt rates, e.g., 2011 to 2012, we average estimates available from January 2011 to December 2012. To assess the uncertainty in melt rate, we assume the errors of the components of the mass balance equation to be uncorrelated, unbiased, and characterized by a Gaussian probability distribution. We obtain an uncertainty ranging from 20 to 30%, or a mean value of 26%.

## Supplementary Material

Appendix 01 (PDF)

## Data Availability

DEMs, interferograms, grounding lines, ice melt rates, error estimates; OMG gravity data data; code have been deposited, respectively, on Dryad (https://doi.org/10.7280/D1XT4G) (61); NASA/JPL/PODAAC-PO.DAAC (https://doi.org/10.5067/OMGEV-BTAG2) (62); and Github (http://github.com/eciraci/ciraci_et_al_2023_petermann) (63).
