# Supplementary material for: Melt rates in the kilometer-size grounding zone of Petermann Glacier, Greenland, before and during a retreat
Source: Proc Natl Acad Sci U S A. 2023 May 8;120(20):e2220924120. doi: 10.1073/pnas.2220924120 (PMC10193949; doi:10.1073/pnas.2220924120)
Supplement: Supplementary file 1 — Appendix 01 (PDF) [file pnas.2220924120.sapp.pdf]

PNAS
www.pnas.org

Supplementary Information for

Melt rates in the kilometer-size grounding zone of Petermann Glacier, Greenland before and during a retreat.

E. Ciracì[a,b], E. Rignot[a,b,c,1], B. Scheuchl[b], V. Tolpekin[d], M. Wollersheim[d], L. An[e,f], P. Milillo[g,h], J. L. Bueso-Bello[h], P. Rizzoli[h], and L. Dini[i]

[a]Jet Propulsion Laboratory, California Institute of Technology, 4800 Oak Grove Drive, Pasadena, CA, USA; [b]University of California, Irvine, Department of Earth System Science, Irvine, CA, USA; [c]University of California, Irvine, Department of Civil and Environmental Engineering, Irvine, CA, USA; [d]ICEYE Oy, Maarintie 6, 02150 Espoo, Uusimaa, Finland; [e]College of Surveying and Geo-informatics, Tongji University, Shanghai, China; [f]Center for Spatial Information Science and Sustainable Development Applications, Tongji University, Shanghai, China; [g]University of Houston, Cullen College of Engineering, Houston, TX, USA; [h]German Aerospace Center (DLR), Microwaves and Radar Institute, Munich, Germany; [i]Italian Space Agency (ASI), Matera, Italy

Email: erignot@uci.edu

This PDF file includes:

Figure S1

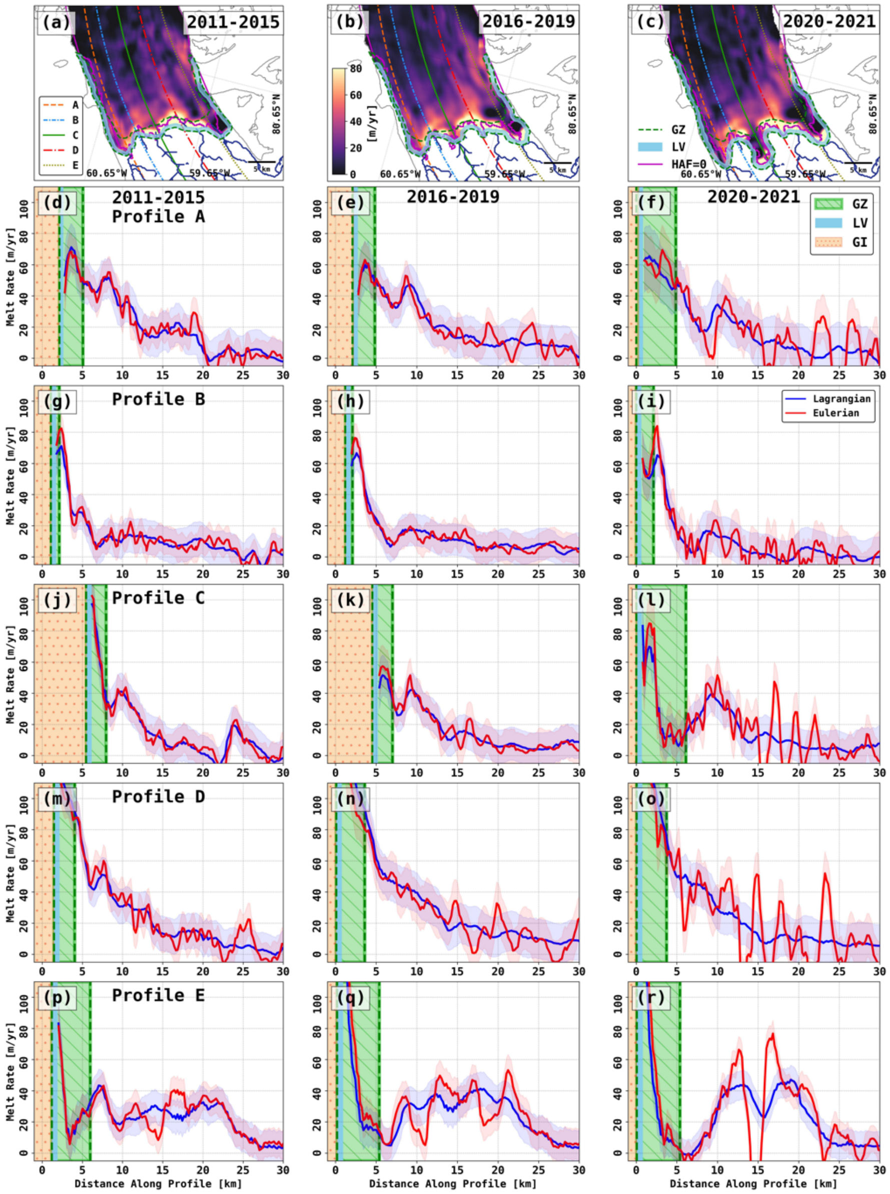

Figure S1. Ice melt rates from a Lagrangian (meters per year, blue; standard error in light blue) and Eulerian frameworks (red, standard error in light red) on the floating ice of Petermann Glacier, Greenland for the years a) 2011-2015; b) 2016-2019; and c) 2020-2021 along profiles A-E (d-r). The green forward diagonal-hatched column in (d-r) indicates the grounding zone (GZ), the orange dotted-hatched column indicates grounded ice (GI), and the light blue band is the Limit of Viability (LV). The location of the profiles is shown color coded in (a). Panel (a-c) show the grounding zone (dotted green), the limit of viability (blue band), where ice crosses the line of flotation (purple), and the location of subglacial channels on grounded ice (black lines).
